# The Physician’s Leisure Library

**Published:** 1891-11

**Authors:** 


THE PHYSICIAN'S LEISURE LIBRARY has given to the profession some excellent articles by standard medical writers. One of the last of the series issued is "Artificial Anaesthesia and Anæsthetics," by Dr. Forrest Willard, M. D., and Dr. Lewis H. Osler, Jr. This will prove a valuable little work for perusal to those who are accustomed to frequently administer anaesthetics.

Too often is the giving of anaesthetics relegated to those whose assistance is thought to be the least needed by the operator, and one perhaps who has had the least experience, when in truth the life of the patient is more dependent upon the skill of the anaesthetizer than to the operator who receives all the glory. The little work while in no ways exhaustive, is very practical, giving many points of importance often overlooked. The Library is published by Geo. S. Davis, Detroit.

